# Occupationally relevant vibrations and the brain: frequency-dependent proteomics signatures in a rat model

**DOI:** 10.1080/07853890.2026.2705692

**Published:** 2026-08-09

**Authors:** Daniel Chao, Stephan Milosavljevic, Brooke Thompson, Lucas Julseth, Paulos Chumala, Megan Tomilin, Angelica Lang, John G. Howland, George S. Katselis

**Affiliations:** ^a^ Health Sciences, College of Medicine, University of Saskatchewan, Saskatoon, Canada; ^b^ Canadian Centre for Rural and Agricultural Health, University of Saskatchewan, Saskatoon, Canada; ^c^ School of Rehabilitation Science, College of Medicine, University of Saskatchewan, Saskatoon, Canada; ^d^ Department of Biochemistry, Microbiology and Immunology, College of Medicine, University of Saskatchewan, Saskatoon, Canada; ^e^ Department of Medicine, College of Medicine, University of Saskatchewan, Saskatoon, Canada; ^f^ Department of Anatomy, Physiology and Pharmacology, College of Medicine, University of Saskatchewan, Saskatoon, Canada

**Keywords:** Whole-body vibration, rats, mass spectrometry-based proteomics, differentially expressed proteins, cognition

## Abstract

**Introduction:**

Occupational exposure to whole-body vibration (WBV), particularly in agricultural environments, has been associated with adverse cognitive and physiological effects. This study examined the neurophysiological impact of WBV in a rat model at 4 Hz and 30 Hz, frequencies representative of off-road and on-road vehicle operation.

**Methodology:**

Forty-four Sprague–Dawley rats were assigned to control (0 Hz), low-frequency (4 Hz), or high-frequency (30 Hz) vibration conditions. After three days of exposure, brain tissues were collected and analyzed using mass spectrometry–based proteomics to identify differentially expressed proteins.

**Results:**

Proteomic profiling revealed distinct, frequency-dependent alterations in brain protein expression. Compared with controls, 32 cognition-related proteins were differentially regulated at 4 Hz and 29 at 30 Hz, with 13 differing between the two vibration conditions. Principal component analysis showed clear separation among groups, indicating unique proteomic signatures for each exposure frequency. Functional enrichment and protein–protein interaction analyses demonstrated involvement of synaptic plasticity, cytoskeletal organization, calcium regulation, and neurotransmitter release. Exposure to 4 Hz was associated with the upregulation of proteins involved in calcium homeostasis and synaptic integrity, suggesting potential disruption of cognitive processes. In contrast, 30 Hz increased the expression of proteins related to axonal guidance and neuroprotection, indicating a less clearly adverse response that may reflect adaptive or potentially beneficial effects.

**Discussion:**

These findings provide new insight into biological mechanisms underlying WBV-induced cognitive changes and underscore the importance of vibration frequency in shaping neurophysiological outcomes. They also establish a foundation for future studies integrating proteomics with behavioural assessments in animals and humans.

## Introduction

Vibration exposure is associated with an increased risk of occupational injury [1]. In agriculture, during typical day-to-day tasks, workers use a variety of farm vehicles, often exposing themselves to high levels of resonant, low-frequency, whole-body vibrations (WBV) that can exceed safe occupational exposure limits [2]. Such exposures contribute to the risk of musculoskeletal disorders, equipment-related injuries (slips, trips and falls), collisions, accidents and possibly death [3]. While the causes of vehicle collisions and accidents are multifactorial, it is estimated that over 85% are related to decrements in human performance [4]. In this context, human performance involves changes within the physical, physiological, cognitive, and emotional domains that will adversely impact task completion, including safe driving. In addition to musculoskeletal disorders, WBV exposure has been shown to negatively impact performance [5] and various performance-related domains, such as decreased attention [6], decreased balance [7,8], increased drowsiness [6,9], and reaction time [10–12]. However, there are gaps in knowledge and understanding regarding how WBV exposure affects cognition, stress, neural pathways, drowsiness, sensation, balance and function [13–17]. Unfortunately, in current agricultural practice, it is not possible to eliminate exposure to WBV, and both the acute and long-term accumulation of vibration exposures and related adverse effects on operator health are of concern. Our fieldwork research has identified high-magnitude, low-frequency (1.3 to 4.9 Hz) resonant axial vibrations in farmers using vehicles during their regular on-farm tasks [18]. Furthermore, various adverse cognitive effects have also been detected at low frequencies, such as decreased sensorimotor processing [19], decreased balance and proprioception [20,21], and decreased attention [22]. Published laboratory research conducted by our team on human participants has also identified acute alterations in cognitive responses following low-frequency vibration exposure [6,12]. Similarly, vibration-induced cognitive decrements in animal models (rats) have also been identified in a small number of published studies exploring motor vehicle driving frequencies [23,24]. However, the vibration frequencies used in these prior experiments were higher than those identified in agricultural studies (30 Hz versus 4-6 Hz, respectively) [2]. To date, we have not identified any published studies that explore evidence of altered levels of brain proteins related to neural disturbances (such as vibration-induced decrements in cognition). Guidelines for vibration exposure exist (ISO 2631-1), which aim to regulate parameters such as vibration magnitude, frequency, and duration. However, they focus on the physical health outcomes of WBV exposure, such as musculoskeletal health outcomes, and do not consider the potential cognitive effects of vibrations. Therefore, a knowledge gap exists regarding how machinery-relevant vibration frequencies adversely affect cognitive function and whether they alter levels of neural biomarkers. Biological studies focused on proteomics will help determine how WBV affects the neurophysiology underlying cognitive performance. Given that many neural proteins are highly conserved between humans and rats, investigating differential protein expression in a rat model will provide valuable insights to predict human outcomes [25]. Using comparative mass spectrometry-based proteomics investigations of brain tissue samples in an animal model following vibration exposure, this research aimed to identify any significant changes in protein expression post-vibration and determine whether these changes are linked to alterations in cognitive performance.

## Methodology

### Experimental design

**Figure 1.**
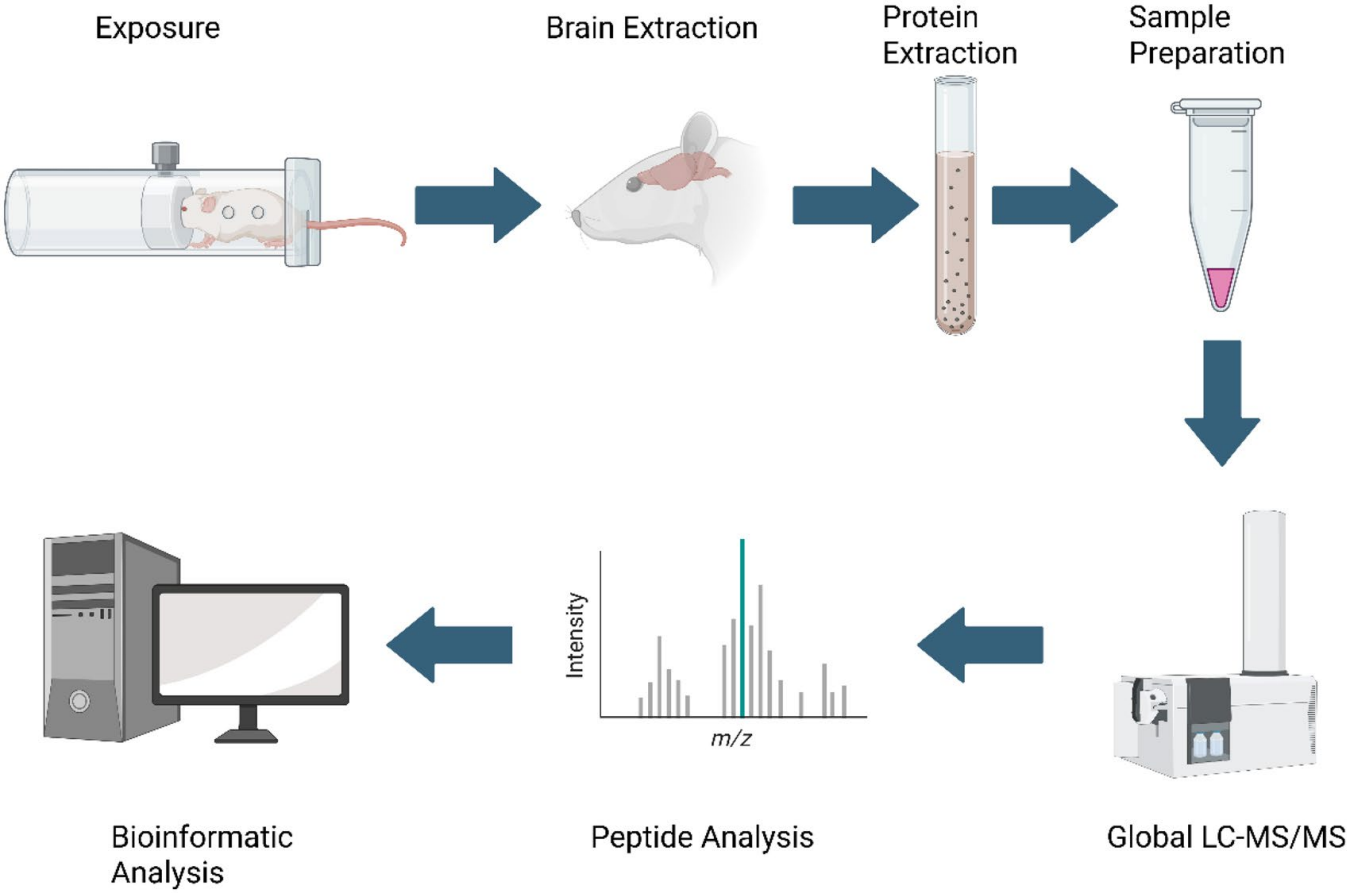
Study flowchart (clockwise). Animals underwent exposure followed by brain extraction, protein extraction, and peptide sample preparation. Peptides were analyzed using global LC–MS/MS, which enabled peptide identification. Differential proteins were then interpreted using bioinformatics analysis.

This study was designed to evaluate the effects of WBV at two occupationally relevant frequencies (4 Hz and 30 Hz) on brain proteomics profiles in a rat model. Adult Sprague-Dawley rats were allocated to one of three exposure conditions: control (0 Hz), low frequency (4 Hz), or high frequency (30 Hz). They then underwent three four-hour days of WBV exposure or control exposure. Following exposure, brains were collected and prepared for analysis using global liquid chromatography tandem mass spectrometry (LC-MS/MS). Results were then subjected to bioinformatics analysis to determine differential protein expression. Researchers were aware of group allocation during exposure delivery but blinded to group allocation during sample preparation. The whole experimental workflow is demonstrated in Figure 1.

### Sprague-Dawley rats (female and male)

Forty-four Sprague-Dawley female (F) and male (M) rats were used (22 of each sex). Females and males weighed 255-265 g and 315-325 g, respectively. Animal experiments were approved by the University of Saskatchewan Animal Research Ethics Board (Animal Use Protocol #20220063) and conducted in accordance with the Canadian Council on Animal Care (CCAC) guidelines. Animals were 8-10 weeks old at the time of experimentation. Animals were housed in pairs and had ad libitum access to food and water. Animals were kept on a 12h:12h light-dark cycle and tested during the light phase. Animals were allowed to habituate for a week before trials and familiarised with the experimental setup through three 10-minute platform habituations within their polyvinyl chloride (PVC) tubes, performed on Friday, Saturday, and Sunday, before vibration exposures began.

**Figure 2.**
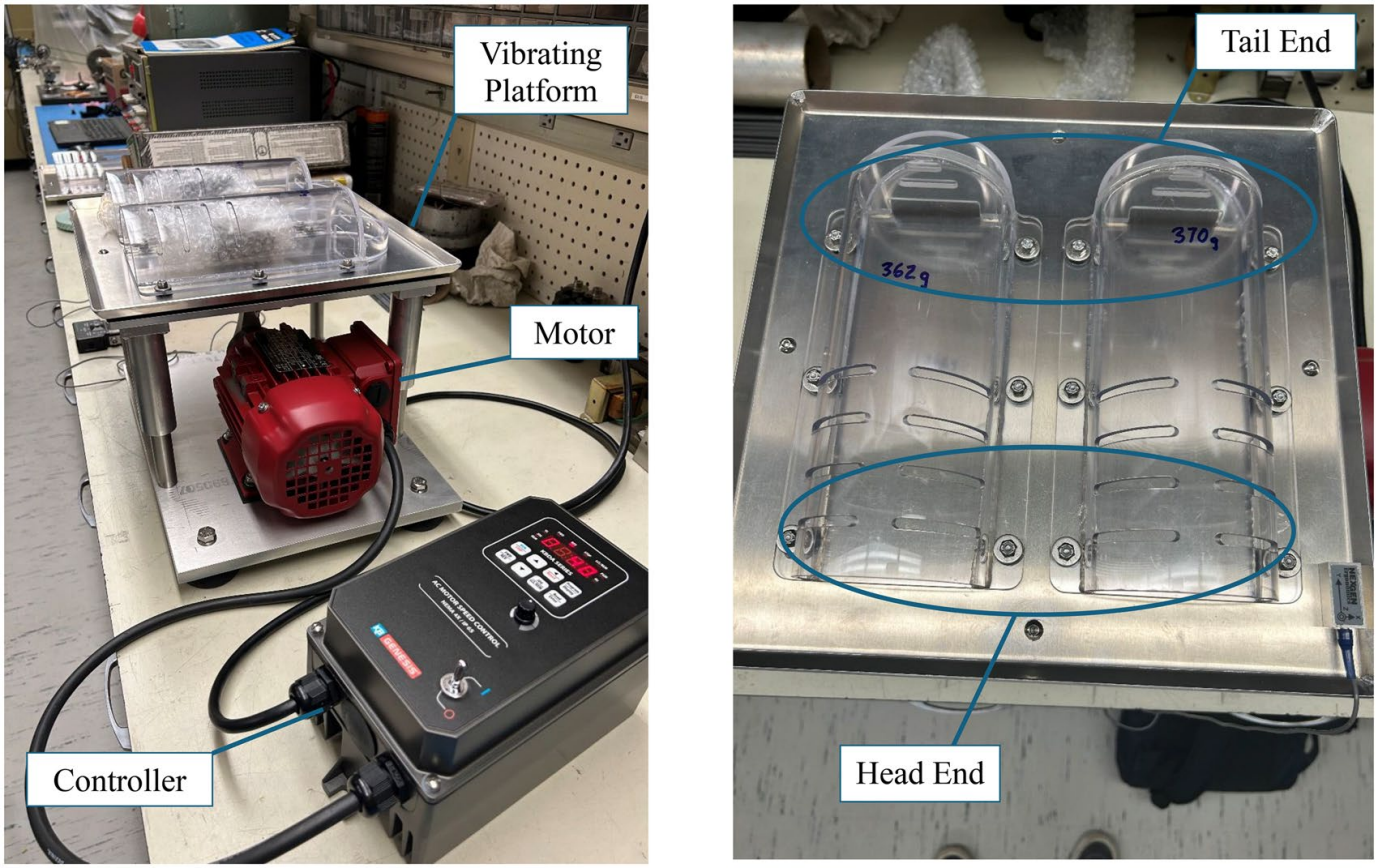
Experimental setup for animal vibration. (a) depicts the exposure platform used to deliver whole-body vibration to rats, including the vibrating platform, motor, and controller. (b) depicts the orientation of animals within PVC restraint tubes during vibration exposure, with the head and tail ends indicated.

Rats were placed individually in a PVC tube mounted on a vibrating platform manufactured in the College of Engineering Machine Shops at the University of Saskatchewan (Figure 2). Each vibrating or non-vibrating platform was fitted with two individual PVC tubes, allowing for two rats to be exposed simultaneously. Rats were allocated to one of three exposure conditions: a) = no-vibration; b) = sinusoidal 4 Hz (0.5 m/s^2^ vertical); or c) = sinusoidal 30 Hz (0.5 m/s^2^ vertical). 4 Hz was selected based on our previous agricultural machinery studies [18], while 30 Hz was selected as the comparator based on published motor vehicle driving studies [26–30]. The number of samples per condition was as follows: 0 Hz: *n* = 16 [F8, M8]; 4 Hz: *n* = 14 [F7, M7]; 30 Hz: *n* = 14 [F7, M7]. Exposure was delivered for four hours daily over three consecutive days (Tuesday, Wednesday, Thursday). The rats were monitored for signs of stress, such as porphyrin staining and excessive grooming [31,32]; however, they showed no observable signs of stress, aside from initial defecation. As no rats displayed any unusual or abnormal behaviour, all brain samples were collected and prepared for analysis.

### Collection of rat brains

Following the three days of exposure, whole-brain samples were collected 24 h later (Friday). Both control and experimental groups were anesthetized using isoflurane gas and then humanely euthanized using carbon dioxide gas. Whole brains were harvested immediately following euthanasia. Incisions were made in their skulls, and the entire brain was collected for proteomics investigation. Brains were flash-frozen in liquid nitrogen and transferred to individual collection tubes for storage at −80 °C. Each biological sample (one brain hemisphere/rat) underwent one preparation (process replicate = 1).

### Tissue homogenization and protein extraction

Frozen brain samples were thawed and immediately sectioned along the midline. The right hemisphere was stored for future studies, while the entire left hemisphere, cerebral and cerebellar regions, was processed for proteomics analysis. Sectioned samples were inserted into Ruptor 7 mL bead tubes, prefilled with 1.4 mm ceramic beads (Omni International) containing RIPA (1X; Thermo Fisher Scientific) extraction buffer and a protease and phosphatase inhibitor cocktail (Thermo Fisher Scientific). Each sample was subjected to two 30-second rounds of bead-beating homogenization, with a 30-second delay between each cycle. Following homogenization, the tubes were placed on ice for 40 min to minimize foaming. Samples were then sonicated in a water bath for 2 min to maximize protein extraction and subsequently placed on ice. Samples were centrifuged at 12,000 rpm for 5 min at 4 °C and then stored at −80 °C until further analysis. Unfortunately, two samples were eliminated during extraction because insufficient protein was extracted from the brain tissues to undergo preparation and analysis. These samples were one female exposed to 30 Hz WBV and one male exposed to 4 Hz WBV. The number of samples proceeding to the next phase of sample preparation was as follows: 0 Hz: *n* = 16 [F8, M8]; 4 Hz: *n* = 13 [F6, M7]; 30 Hz: *n* = 13 [F7, M6].

### Protein digestion

Prior to digestion, protein was quantified using the Pierce BCA Protein Assay Kit (Thermo Fisher Scientific) according to the manufacturer’s protocols. All samples were normalized to 2 mg/mL. In-solution digestion was performed using a protocol developed in our laboratory [33,34]. Proteins were denatured using 50% trifluoroethanol (Fisher Scientific), followed by reduction with 10 mM dithiothreitol (MP Biomedicals) at 60 °C for 1 h at 300 rpm (Eppendorf Thermomixer). To prevent reformation of disulphide bonds, 50 mM iodoacetamide (Fisher Scientific) was added, and samples were incubated in the absence of light at 37 °C for 30 min at 500 rpm. Solvents were then evaporated using a SpeedVac (Labconco) and samples were incubated with cold acetone at −80 °C for 1 h. Samples were then centrifuged at 13,000 rpm for 30 min at 4 °C (Eppendorf 5430 R). The acetone supernatant was discarded and replaced with fresh cold acetone, followed by another incubation period at −80 °C for 1 h and centrifugation at 13,000 rpm for 30 min at 4 °C. The supernatant was then removed *via* SpeedVac evaporation. The resulting precipitate was resuspended using 100 mM ammonium bicarbonate (ABC; Fisher Scientific). Trypsin/Lys-C buffer (50 ng/μL Trypsin/Lys-C in 0.1% acetic acid and 200 mM ABC; Pierce) was added at a 1:40 enzyme-to-protein ratio, and samples were digested overnight at 37 °C and 300 rpm. Samples were then evaporated using a SpeedVac and stored at −80 °C.

### Desalting via zip-tipping

Samples were desalted using C18 Omix tips (Agilent Technologies) using an in-house protocol [33,34]. Specifically, C18 tips were primed sequentially using 100% acetonitrile (ACN), 50% ACN and 50% water (H_2_O), then 0.1% trifluoroacetic acid (TFA) in H_2_O. Samples were reconstituted in a 3% ACN, 97% MS-grade H_2_O solution, and then adjusted to pH 3 using 2.5% TFA. Samples were loaded onto preconditioned tips by repeated aspiration and dispensing, then washed out with 0.1% TFA in MS-grade H_2_O. Bound peptides were eluted from the C18 tips sequentially through repeated aspiration and dispensing with 60% ACN/0.1% formic acid (FA) and 70% ACN/0.1% FA. Eluates were combined, evaporated using the SpeedVac, and then stored at −80 °C awaiting proteomics analysis.

### LC-MS/MS

Before mass spectrometric analysis, peptide samples were reconstituted in 30 μL of buffer [97:3:1, v/v/v; MS-grade H_2_O, ACN, and formic acid (HF); Fisher Scientific] and transferred to Agilent mass spectrometry vials. Comparative proteomics analyses were performed on an Agilent 6550 iFunnel quadrupole time-of-flight (QTOF) instrument equipped with an Agilent 1260 series liquid chromatography system and an Agilent Chip Cube LC–MS interface (Agilent Technologies) [33]. Chromatographic separations were achieved using a high-capacity Agilent HPLC Chip II: G4240–62030 Polaris-HR-Chip_3C18 containing a 360 nL enrichment column and a 75 μm × 150 mm analytical column, both packed with Polaris C18-A resin (180 Å, 3 μm stationary phase). Samples (2 μL) were first loaded onto the enrichment column with 0.1% HF in H_2_O at a flow rate of 2.0 μL/min, then transferred onto the analytical column. Peptides were separated with a linear gradient of solvent A (0.1% HF in H_2_O) and solvent B (0.1% formic acid in ACN). The linear gradient was 3–25% B over 105 min, 25–40% B over 15 min, 40–90% B over 5 min, and held at 90% B for 5 min, at a flow rate of 0.3 μL/min. Two LC–MS/MS injections (technical replicate = 2) were performed and were averaged before statistical analysis.

Positive ion electrospray ionization was performed with a capillary voltage of 1900 V, an ion fragmentor of 360 V, and nitrogen drying gas of 225 °C with a flow rate of 12 L/min. MS survey spectra were collected across a mass range of m/z 250–1700 at a scan rate of 8 spectra/s. MS/MS spectra were acquired across m/z 100–1700 with an isolation width of 1.3 amu.

### Bioinformatics analysis

The raw mass spectrometry data obtained from LC-MS/MS were extracted using the Spectrum Mill extractor (Agilent Technologies) with the following parameters - fixed modification: cysteine carbamidomethylation; precursor MH^+^: 300 to 8000 Da; scan time range: 0 to 300 min; sequence tag length > 0; retention time (RT): ± 120, m/z tolerance: ± 0.05 m/z; precursor selection purity and spectral similarity as well as RT and m/z merging constraints; merge MS^2^ and MS3 spectra from same precursor; and 500 counts MS noise threshold.

Extracted data was then searched against the SwissProt *Rattus Norvegicus* database (February 2025) using the Spectrum Mill search engine (Agilent Technologies). The search strategy consisted of 6 search steps, with 2 auto-validation steps between each. The search parameters that were fixed among all steps were as follows: 2 missed enzyme cleavages, monoisotopic masses; ± 20 ppm precursor mass tolerance; ± 50 ppm product mass tolerance; fixed modifications, cysteine carbamidomethylation. For variable modifications, the following steps in the search were used: Step 1 parameters selected Trypsin/Lys-C as the enzyme digest, and carbamylated lysine, oxidized methionine, pyroglutamic acid, deamidated asparagine and phosphorylated serine, threonine, and tyrosine. Step 2 parameters selected Trypsin/Lys-C as the enzyme digest and the same variable modifications as in Step 1, except that acetylated lysine was chosen instead of carbamylated lysine. The third search step selected trypsin non-specific C-terminus as the enzyme digest. The fourth search step selected trypsin as the non-specific N-terminus enzyme digest. The fifth search step selected Lys-C non-specific C-terminus as the enzyme digest. The sixth search step selected Lys-C as the non-specific N-terminus enzyme digest. Auto-validation was performed between each searching step and consisted of two steps.

The first auto-validation step selected peptide mode, a maximum false discovery rate (FDR) of 1%, and a minimum sequence length of 6. The second step of auto validation selected the protein polishing mode, grouped proteins across all directories, and set FDR at 1%.

### Statistical rationale

Statistical analyses of our results were performed using Mass Profiler Professional software (Agilent Technologies). Analysis was performed based on the total spectral intensity of the proteins by summing the areas for each validated peptide. Spectral intensities were normalized based on protein concentration using an external scalar. The external scalar was calculated by dividing the protein concentration of each sample by that of the lowest sample, as determined by protein quantification. Two parameters were used to evaluate our data: sex (female versus male) and exposure (0, 4, and 30 Hz). Normalized proteins were then filtered based on their abundance and frequency (i.e. they must be present in all samples for at least one condition). Intensities were transformed using log_2_, and a two-way ANOVA with a multiple hypothesis testing correction was performed. Benjamini controlled multiple testing–Hochberg FDR and a list of statistically significant proteins (*p* < 0.001) with a fold change (FC) ≥ 10 was created. The stringent thresholds of *p* < 0.001 and FC ≥ 10 were employed to ensure high confidence in our results [33,35,36], thereby strengthening the validity of our findings.

Principal component analysis (PCA) was performed based on the differentially regulated proteins identified. The SwissProt database (www.uniprot.org) was used to determine the functions of differentially regulated proteins. STRING analysis (https://string-db.org/) was used to identify gene ontology (GO) categories of differentially regulated proteins and to identify protein-protein interactions.

## Results

### Protein identification

**Figure 3.**
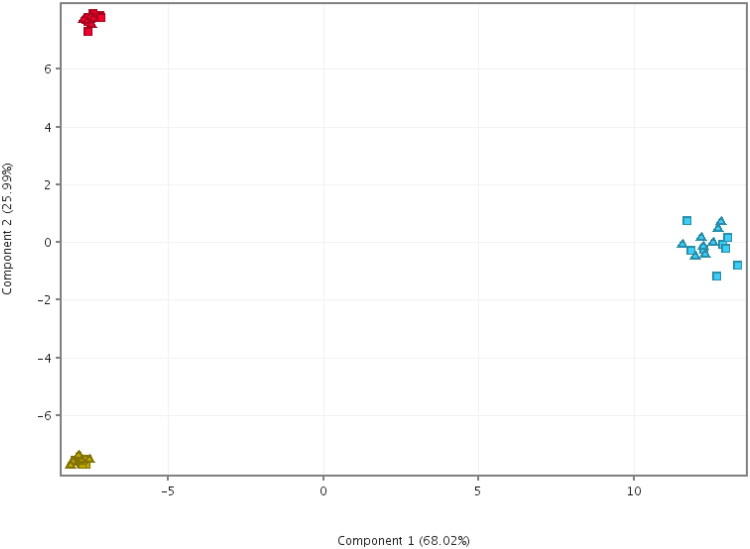
Principal Component Analysis (PCA) of proteomics profiles from rat brain tissue following whole-body vibration. PCA plot visualizes the global variance in protein expression among three experimental groups: control (0 Hz), 4 Hz, and 30 Hz vibration.

After searching the SwissProt *Rattus Norvegicus* database, we identified 5600 proteins cumulative for all samples. Following this identification, bioinformatics analysis was performed, and 106 proteins were identified as differentially expressed between the 4 Hz and control conditions, 110 between the 30 Hz and control conditions, and 52 between the 4 Hz and 30 Hz conditions. Principal component analysis of the differentially identified proteins (Figure 3) revealed distinct separation among the three experimental groups, indicating clear differences in the brain proteome following these vibration exposures. However, sex showed no observable impact on overall protein expression in any experimental group. These results suggest that the differential protein regulation in response to WBV is frequency dependent.

### Functional protein analysis

Differentially expressed proteins were analyzed using GO enrichment to cluster proteins based on their associated biological functions (Figure 4). Figure 4A (4 Hz versus 0 Hz) indicates a high enrichment in cellular biological processes, including localization, intracellular transport, and vesicle-mediated transport, indicating broad roles in maintaining neurons and regulating intracellular trafficking. Figure 4B (30 Hz versus 0 Hz) illustrates enrichment in cellular biological processes related to cell cycle regulation, localization and cellular organization, indicating broad roles in neuronal remodelling and the structural maintenance of neurons. Figure 4C (4 Hz versus 30 Hz) demonstrates enrichment in cellular biological processes related to development, regulation of neurotransmitters, and SNARE protein complex assembly. These findings suggest that many of the differentially expressed proteins collectively converge on pathways related to synaptic function, neural plasticity, and cognitive regulation.

Figure 4.Gene ontology outlining the biological processes of the identified differentially expressed proteins. This figure depicts the gene ontology biological process enrichment analysis for the differentially expressed proteins identified in the 0 Hz vs 4 Hz (A), 0 Hz vs 30 Hz (B), and 4 Hz vs 30 Hz (C) conditions. The position of each bubble on the y-axis indicates the specific biological process, while its position on the x-axis indicates the enrichment signal, with bubbles farther to the right representing stronger enrichment. Bubble size corresponds to the number of differentially expressed proteins associated with each biological process, with larger bubbles indicating a greater number of proteins. Bubble colour represents the false discovery rate (FDR), with darker colours indicating lower FDR values and therefore greater statistical significance. Graphs were generated using STRING (https://string-db.org/).

**Figure:**
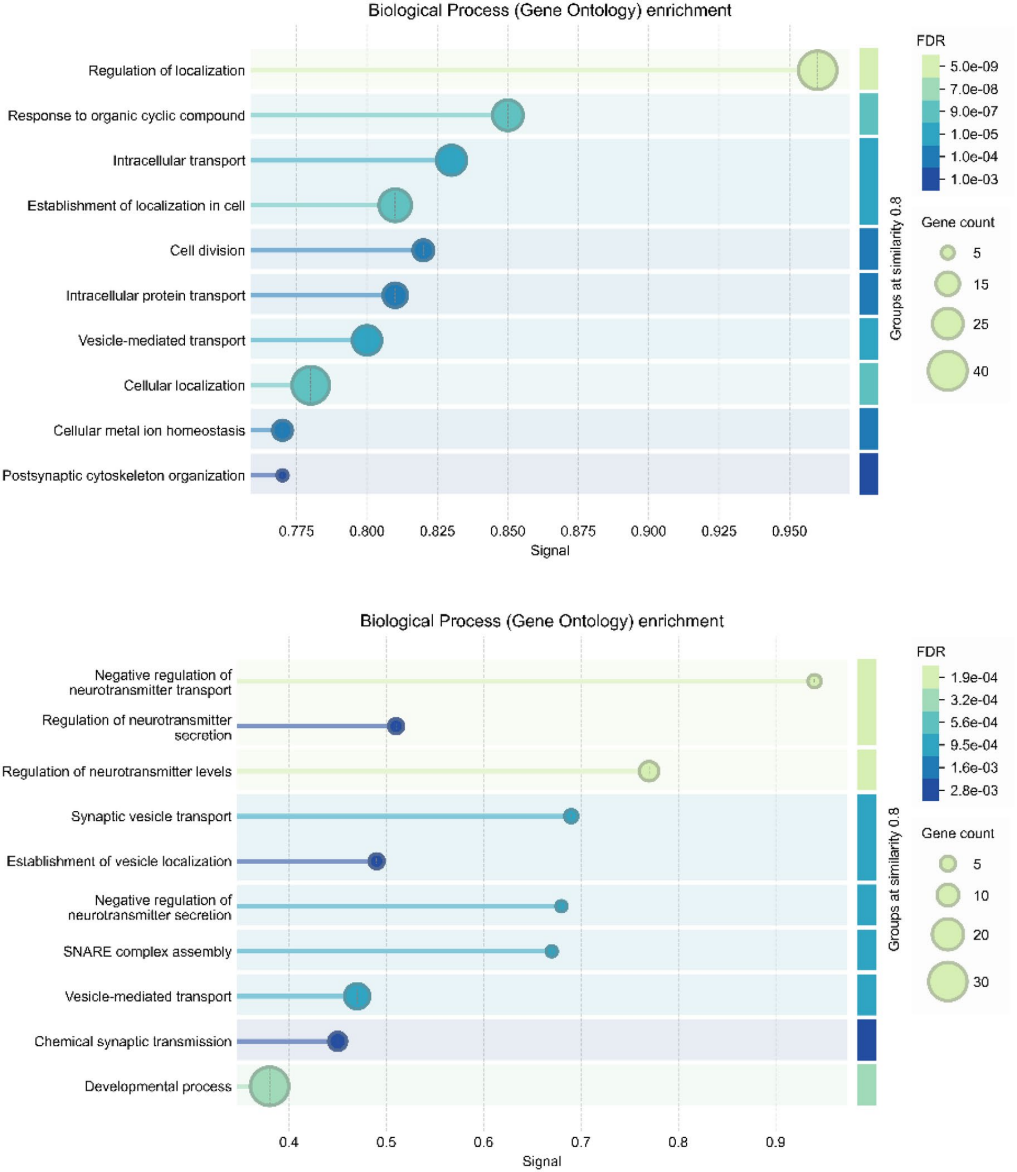

**Figure:**
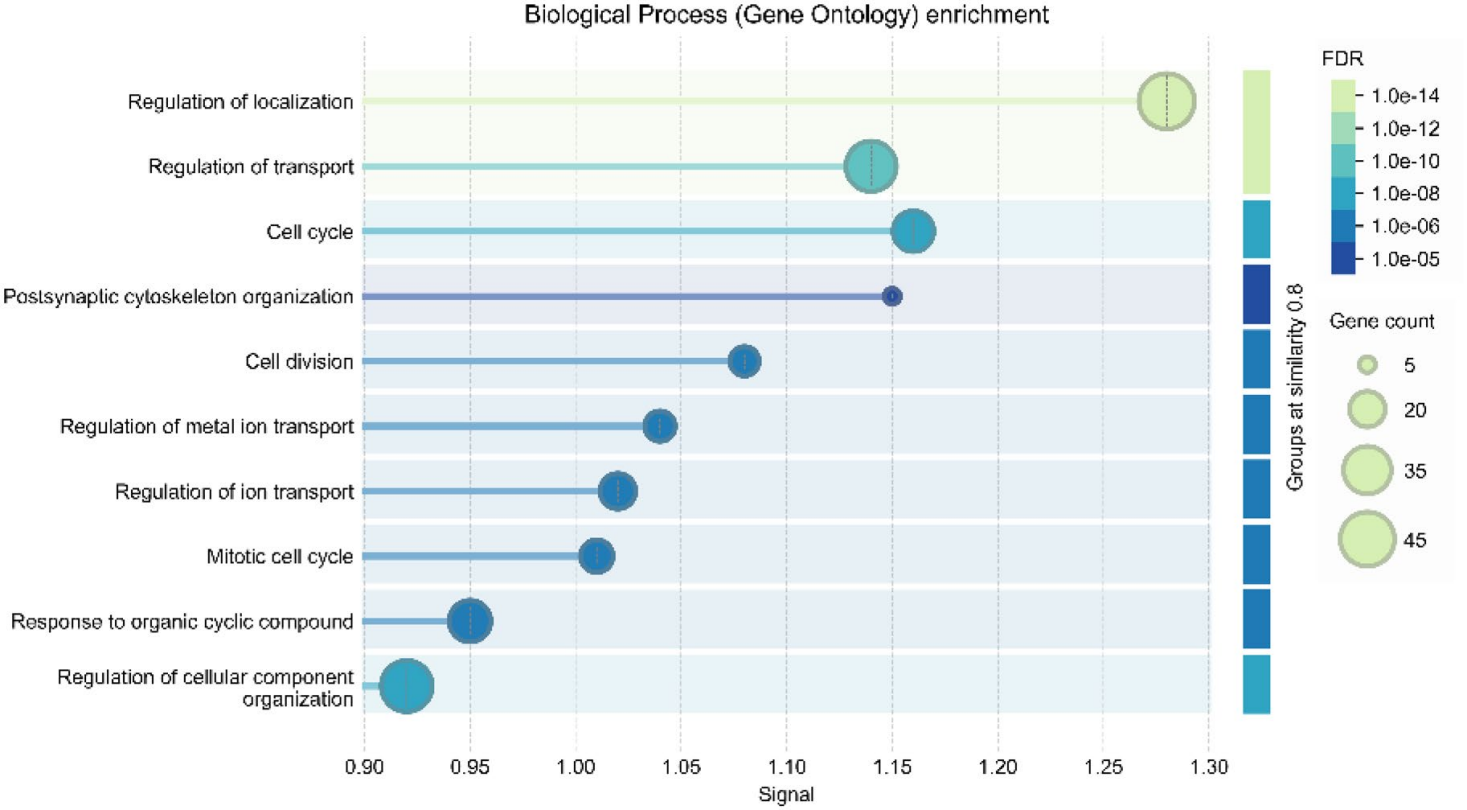

Through functional analysis using www.uniprot.org, we identified, by consensus, differentially expressed proteins associated with neuronal processes linked to cognition. Tables 1–3 (*with accession numbers, homology to human proteins, and their known functions)* list 32 proteins identified between 4 Hz and 0 Hz, 29 between 30 Hz and 0 Hz, and 13 between 4 Hz and 30 Hz. These proteins were predominantly upregulated between 4 Hz and 0 Hz, and between 30 Hz and 0 Hz, while those between 4 Hz and 30 Hz displayed a balanced mix of upregulated and downregulated proteins.

**Table 1.** List of proteins identified to be involved in neuronal processes relevant to cognition between 4 Hz and 0 Hz.

Protein Name	Rat Accession Number	Human Accession Number	Regulation (4 Hz vs 0 Hz)	Log FC (4 Hz vs 0 Hz)	Function^a^
Microtubule-associated protein 1B	P15205	P46821	Up	24.3	Microtubule regulator essential for neurite outgrowth, axon guidance, and synapse maturation.
Microtubule-associated protein RP/EB family member 2	Q3B8Q0	Q15691	Up	20.8	Tracks and organizes microtubules; supports cytoskeletal remodelling and neuronal development.
Neurofilament heavy polypeptide	P16884	P12036	Up	22.1	Intermediate filament for axonal stability; controls axon calibre and conduction; linked to neurodegeneration.
Neurofilament medium polypeptide	P12839	P07197	Up	23.3	Intermediate filament subunit maintaining axonal structure; regulates axon diameter and conduction.
Plasma membrane calcium-transporting ATPase 1	P11505	P20020	Up	21.6	Plasma-membrane Ca^2+^ efflux pump; maintains neuronal Ca^2+^ homeostasis and supports synaptic transmission.
Plasma membrane calcium-transporting ATPase 3	Q64568	Q16720	Up	21.3	Presynaptic Ca^2+^ efflux pump for recovery and plasticity; variants linked to intellectual disability.
Ras-related protein Rab-14	P61107	P61106	Up	21.9	Endocytic/recycling Rab GTPase; supports neurite formation.
Ras-related protein Rab-6A	Q9WVB1	P20340	Up	20.9	Golgi-to-ER retrograde Rab coordinates neurite and axon outgrowth.
Septin-11	B3GNI6	Q9NVA2	Up	22.8	Cytoskeletal GTPase shaping dendrites/spines and organizing GABAergic synapses.
Septin-4	A0A096MJN4	O43236	Up	20.5	Presynaptic scaffold modulating dopaminergic signalling; alterations linked to Parkinsonism.
Septin-8	B0BNF1	Q92599	Up	21.5	Facilitates the assembly of the SNARE complex in synaptic vesicle trafficking.
Serine/threonine-protein phosphatase PP1-alpha catalytic subunit	P62138	P62136	Up	20.5	Ser/Thr phosphatase forming diverse holoenzymes; regulates transcription and long-term synaptic plasticity.
Synapsin-2	Q63537	Q92777	Up	25.0	Synaptic-vesicle phosphoprotein that tethers vesicles and modulates neurotransmitter release.
Synaptotagmin-2	P29101	Q8N9I0	Up	21.3	Membrane-interacting vesicle protein; participates in synaptic vesicle trafficking and exocytosis.
Syntaxin-1A	P32851	Q16623	Up	21.7	SNARE protein mediating synaptic vesicle fusion and endocytosis; essential for neurotransmitter release.
14-3-3 protein epsilon	P62260	P62258	Up	25.4	Adaptor protein modulating many signalling pathways; mediates nuclear/ cytoplasmic shuttling (e.g. HSF1).
14-3-3 protein eta	P68511	Q04917	Up	23.8	Adaptor protein regulating diverse signalling pathways.
14-3-3 protein gamma	P61983	P61981	Up	25.0	Adaptor in diverse signalling; reported interactions include GATOR2 complex and NMDA receptor pathways.
14-3-3 protein theta	P68255	P27348	Up	24.2	Adaptor protein regulating diverse signalling pathways.
Alpha-internexin	P23565	Q16352	Up	24.4	Neuronal intermediate filament supporting neurite morphogenesis and axonal integrity.
Beta-synuclein	Q63754	Q16143	Up	23.4	Synaptic protein with neuroprotective and anti-apoptotic function.
Calcium/calmodulin-dependent protein kinase type II subunit beta	P08413	Q13554	Up	23.1	Protein involved in dendritic spine synapse formation and neuronal plasticity.
Calcium/calmodulin-dependent protein kinase type II subunit gamma	P11730	Q13555	Up	20.5	Protein involved in dendritic spine synapse formation and neuronal plasticity.
Cell division control protein 42 homolog	Q8CFN2	P60953	Up	22.4	Rho GTPase modulating dendritic spine plasticity and long-term signalling.
Creatine kinase M-type	P00564	P06732	Up	21.0	Cytosolic phosphotransferase is essential in energy transduction in the brain.
Creatine kinase U-type, mitochondrial	P25809	P12532	Up	22.5	A mitochondrial phosphotransfer enzyme involved in energy transduction in the brain.
Dihydropyrimidinase-related protein 1	Q62950	Q14194	Up	24.0	A protein involved in semaphoring signalling, cytoskeleton remodelling, and axon guidance.
Dihydropyrimidinase-related protein 3	Q62952	Q14195	Up	22.5	A protein involved in semaphoring signalling, cytoskeleton remodelling, and axon guidance.
Endoplasmin	Q66HD0	P14625	Up	23.8	ER chaperone modulating Wnt pathway components.
Gamma-aminobutyric acid receptor subunit gamma-2	P18508	P18507	Down	−14.9	A subunit of GABA channels, which are essential for proper nerve transmission.
Glutamate receptor 2	P19491	P42262	Down	−14.5	AMPA receptor subunit mediating fast excitatory synaptic currents.
KH domain RNA-binding protein 3	Q9JLP1	O75525	Down	−17.7	RNA-binding protein involved in the regulation of alternative splicing.

^a^Protein functions were determined through uniprot.org.

*Note:* The proteins listed above are differentially regulated in 4 Hz conditions compared to 0 Hz. Noted within this table are the names of the proteins, rat and human accession numbers, fold change, and function.

Abbreviations: Hz, hertz; Log FC, logarithm of base 2-fold change; ER, endoplasmic reticulum; Ca^2+^, calcium; ATP, adenosine triphosphate; ADP, adenosine diphosphate; GTP, guanosine triphosphate; RP/EB, retinitis pigmentosa/end-binding protein family; RNA, ribonucleic acid; BDNF, brain-derived neurotrophic factor.

**Table 2.** List of proteins identified to be involved in neuronal processes relevant to cognition between 30 Hz and 0 Hz.

Protein Name	Rat Accession Number	Human Accession Number	Regulation (30 Hz vs 0 Hz)	Log FC (30Hz vs 0 Hz)	Function^a^
**ADP/ATP translocase 2**	Q09073	P05141	up	25.0	Plays a central role in energy transduction in the brain
**ATP-dependent 6-phosphofructokinase, muscle type**	P47858	P08237	up	23.6	Rate-limiting glycolytic enzyme involved in energy supply. Involved in synaptic plasticity, learning, and the development of neurons.
**Cyclin-dependent kinase 2**	Q63699	P24941	down	−15.2	Cell cycle kinase that is involved in neurite, dendritic spine, and synapse formation and is engaged in long-term potentiation and learning.
**Epsin-2**	Q9Z1Z3	O95208	down	−14.0	Adaptor protein involved in clathrin-mediated endocytosis and morphogenesis of neurons.
**Epsin-3**	Q4V882	Q9H201	up	17.0	Adaptor protein involved in clathrin-mediated endocytosis and morphogenesis of neurons, and also involved in energy transduction.
**L-lactate dehydrogenase B chain**	P42123	P07195	up	25.5	Adapter protein implicated in the regulation of many signalling pathways and also involved in energy metabolism.
**Murinoglobulin-1**	Q03626	N/A	up	21.9	Adapter protein implicated in the regulation of many pathways. Modulates inflammation and extracellular protease activity.
**Nucleoside diphosphate kinase A**	Q05982	P15531	up	22.2	Generates NTPs from NDPs; participates in signalling and synaptic plasticity.
**Nucleosome assembly protein 1-like 1**	Q9Z2G8	P55209	up	19.0	Histone chaperone for nucleosome assembly/disassembly; influences transcription and neurogenesis.
**Septin-4**	A0A096MJN4	O43236	up	20.3	Necessary for semaphorin signalling, involved in axon guidance and dopamine signalling.
**Serine/threonine-protein phosphatase 2 A 55 kDa regulatory subunit B alpha isoform**	P36876	P63151	down	−12.1	Involved in neuronal signalling and plasticity.
**Synaptotagmin-2**	P29101	Q8N9I0	up	21.1	Calcium sensor for fast neurotransmitter release at presynaptic terminals.
**Tubulin alpha-4A chain**	Q5XIF6	P68366	up	24.9	Involved in cell division, regulates cell responses, cell migration, formation of filopodia, and phagocytosis.
**14-3-3 protein beta/alpha**	P35213	P31946	up	23.8	Required for dopaminergic metabolism and modulates synaptic function.
**14-3-3 protein epsilon**	P62260	P62258	up	25.2	Phosphoserine adaptor protein supporting neuronal migration, axon outgrowth, and plasticity. Participates in the SNARE complex..
**14-3-3 protein eta**	P68511	Q04917	up	23.6	Phosphoserine adaptor protein contributing to dendritic architecture and GABAergic connectivity.
**Creatine kinase U-type, mitochondrial**	P25809	P12532	up	22.3	A mitochondrial phosphotransfer enzyme involved in energy transduction in the brain.
**Dihydropyrimidinase-related protein 3**	Q62952	Q14195	up	22.5	A protein involved in semaphoring signalling, cytoskeleton remodelling, and axon guidance.
**Dynein light chain 2, cytoplasmic**	Q78P75	Q96FJ2	up	20.6	Component of the dynein motor protein. Supports transport and synaptic maturation.
**Elongation factor 1-alpha 2**	P62632	Q05639	up	23.0	Translation elongation factor; also binds actin to modulate spine structure.
**Endoplasmic Reticulum Chaperone BiP**	P06761	P11021	up	23.5	Chaperone protein mediating protein folding and the unfolded protein response.
**Fructose-bisphosphate aldolase C**	P09117	P09972	up	25.3	Glycolytic aldolase enzyme involved in energy metabolism.
**Heat shock protein HSP 90-beta**	P34058	P08238	up	24.1	Neuronal protein involved in multiple functions such as synaptic signalling and proteostasis.
**Isocitrate dehydrogenase [NADP] cytoplasmic**	P41562	O75874	up	22.4	Important for producing NADPH for redox balance.
**KH domain-containing, RNA-binding, signal transduction-associated protein 1**	Q91V33	Q07666	down	−11.5	RNA-binding adaptor protein involved in synaptic signalling and plasticity.
**Microtubule-associated protein 1B**	P15205	P46821	up	24.1	Microtubule regulator essential in plasticity, neurite outgrowth, axon guidance, and necessary for learning.
**Serine/threonine-protein phosphatase 2 A 55 kDa regulatory subunit B delta isoform**	P56932	Q66LE6	up	18.4	Phosphoprotein involved in neurotransmitter release and neuronal signalling.
**Sodium/potassium-transporting ATPase subunit alpha-2**	P06686	P50993	up	24.9	The sodium/potassium-transporting ATPase subunit alpha-2 is a calcium ion pump essential for maintaining ionic gradients and supporting excitability and neurotransmission.
**Tubulin beta-2B chain**	Q3KRE8	Q9BVA1	down	−10.2	Protein required for neurite and axonal transport.

**^a^**Protein functions were determined through uniprot.org.

*Note.* The proteins listed above are differentially regulated in 30 Hz conditions compared to 0 Hz. Noted within this table are the names of the proteins, rat & human accession numbers, fold change, and function.

Abbreviations: Hz, hertz; Log FC, logarithm of base 2 fold change; ER, endoplasmic reticulum; Ca^2+^, calcium; ATP, adenosine triphosphate; ADP, adenosine diphosphate; GTP, guanosine triphosphate; RP/EB, retinitis pigmentosa/end-binding protein family; RNA, ribonucleic acid; BDNF, brain-derived neurotrophic factor.

**Table 3.** List of proteins identified to be involved in neuronal processes relevant to cognition between 30 Hz and 4 Hz.

Protein Name	Rat Accession Number	Human Accession Number	Regulation (30 Hz vs 4 Hz)	Log FC (30 Hz vs 4 Hz)	Function^a^
Microtubule-associated protein RP/EB family member 2	Q3B8Q0	Q15691	up	20.9	Promotes neurite outgrowth and microtubule reorganization.
**Syntaxin-1A**	P32851	Q16623	up	23.3	Presynaptic modulator of SNARE for vesicle fusion. Enables fast neurotransmitter release and plasticity.
**Alpha-synuclein**	P37377	P37840	down	−24.6	Involved in synaptic activity and SNARE assembly.
**Beta-synuclein**	Q63754	Q16143	up	24.8	Synaptic protein supporting plasticity.
**Dihydropyrimidinase-related protein 4 (Fragment)**	Q62951	O14531	down	−24.6	Necessary for semaphorin signalling, involved in axon guidance.
**Glutamate receptor 2**	P19491	P42262	down	−4.4	AMPA receptor subunit mediating fast excitatory synaptic currents.
**KH domain-containing, RNA-binding, signal transduction-associated protein 1**	Q91V33	Q07666	up	20.0	RNA-binding adaptor protein involved in synaptic signalling and plasticity.
**KH domain-containing, RNA-binding, signal transduction-associated protein 3**	Q9JLP1	O75525	down	−19.9	RNA-binding protein involved in the regulation of alternative splicing.
**Neurabin-1**	O35867	Q9ULJ8	up	18.7	Actin-binding scaffold protein involved in linking the cytoskeleton and the synaptic junction.
**Protein NDRG4**	Q9Z2L9	Q9ULP0	down	−21.5	Neuronal differentiation factor supporting BDNF levels and neuronal survival
**Ras-related protein Rap-1A**	P62836	P62834	up	21.8	A small GTPase that promotes neurite outgrowth and counteracts Ras mitogenic signalling.
**Transforming protein RhoA**	P61589	P61586	down	−22.5	Rho GTPase is involved in regulating various cellular responses, including neurite morphology.
**Translation initiation factor eIF2 assembly protein**	Q62834	Q9BY44	up	18.8	Assembly factor for the eIF2 complex, involved in the initiation of translation.

**^a^**Protein functions were determined through UniProt.org.

*Note.* Proteins listed above are differentially regulated in 30 Hz conditions with respect to 4 Hz. Noted within this table are the names of the proteins, rat & human accession numbers, fold change, and function.

Abbreviations: Hz, hertz; Log FC, logarithm of base 2 fold change; ER, endoplasmic reticulum; Ca^2+^, calcium; ATP, adenosine triphosphate; ADP, adenosine diphosphate; GTP, guanosine triphosphate; RP/EB, retinitis pigmentosa/end-binding protein family; RNA, ribonucleic acid; BDNF, brain-derived neurotrophic factor.

Among the proteins listed in Table 1 are calcium/calmodulin-dependent protein kinase type II subunit beta (CaMKIIβ), glutamate receptor 2 subunit of the AMPA receptor (GluA2), and 14-3-3 protein gamma (14-3-3γ). They have established key functions involved in cognition and are associated with neural processes such as long-term potentiation and memory consolidation. The proteins in Table 2 include 14-3-3 protein epsilon (14-3-3ε), microtubule-associated protein 1B (MAP1B), and synaptotagmin-2 (SYT2), which are considered key regulators of cognition with strong links to neuronal development, axonal guidance, and calcium-triggered neurotransmitter release. In Table 3, GluA2 and synaptotagmin-2 (SYT2) are directly linked to cognition. These proteins are crucial for neurotransmitter release and the processing of short-term memory.

Using STRING analysis, Figure 5 shows protein-protein clustering of proteins impacting cognition across the three exposure groups. Figures 5A and 5B display high connectivity between synaptic and cytoskeletal proteins, such as GluA2 and CaMKIIβ (4 Hz v 0 Hz), MAP1B (30 Hz v 0 Hz), and members of the 14-3-3 protein family (both). These networks highlight roles in synaptic signalling, structural integrity, and plasticity. Figure 5C (30 Hz v 4 Hz) displays less intricate and connected clusters; however, interactions between syntaxin-1A, RhoA, and KH domain-containing proteins illustrate their link to neurotransmitter release.

Figure 5.Protein-protein interactions of cognition-relevant differentially regulated proteins. (A) illustrates the protein-protein interactions of cognition-relevant proteins that are differentially regulated between 4 Hz and 0 Hz. (B) illustrates the protein-protein interactions of cognition-relevant proteins that are differentially regulated between 30 Hz and 0 Hz. (C) illustrates the protein-protein interactions of cognition-relevant proteins that are differentially regulated between 30 Hz and 4 Hz.

**Figure:**
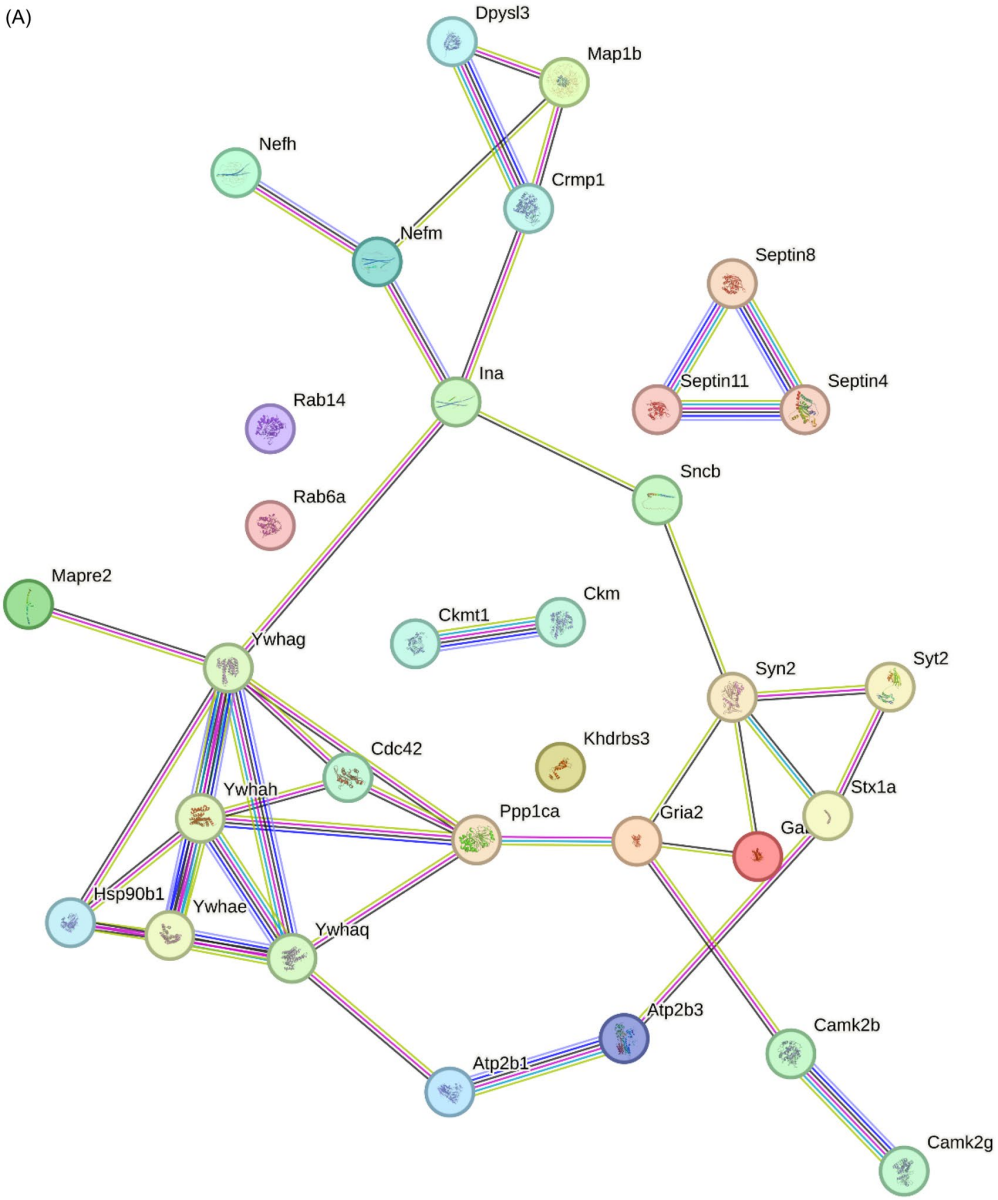

**Figure:**
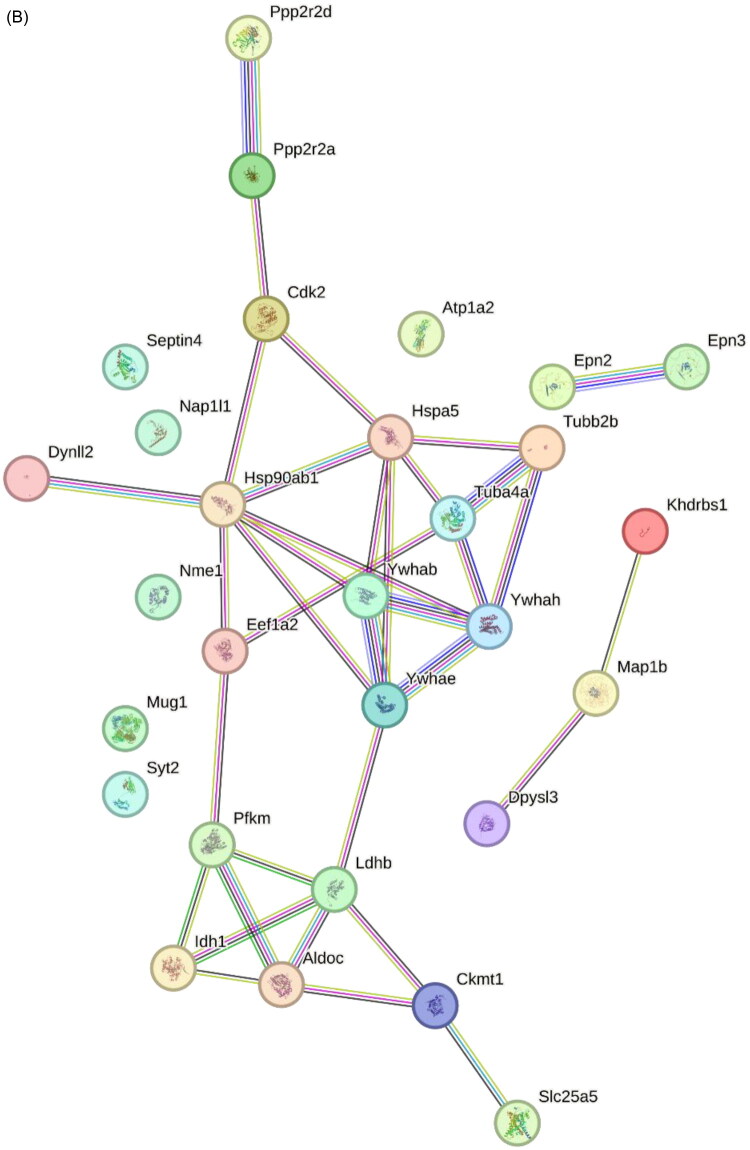

**Figure:**
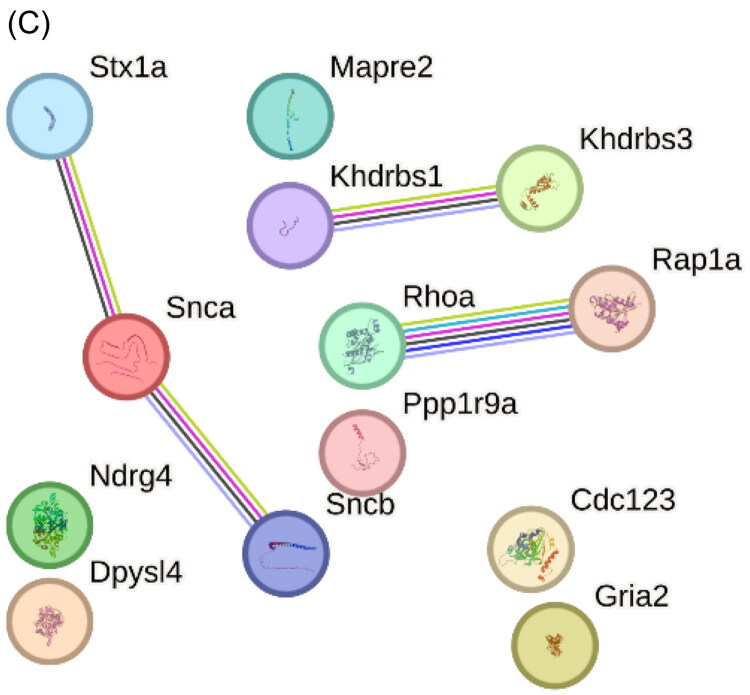

## Discussion

In a rat model, we used mass spectrometry-based proteomics to investigate the potential effects of occupationally relevant WBV frequencies (4, 30, and 0 Hz) on cognition. These analyses revealed 32 cognition-related proteins that were differentially regulated at 4 Hz compared to 0 Hz, 29 at 30 Hz compared to 0 Hz, and 13 cognition-related proteins that were differentially regulated at 30 Hz compared to 4 Hz. All of these cognitively relevant proteins were homologous to those in humans, thus demonstrating the classical mammalian link, in which essential neural pathways and proteins are highly conserved between rodents and humans [15]. Most changes occurred in the broad functional pathways of synaptic plasticity, cytoskeletal organization, and neurotransmitter regulation [37–40]. PCA results revealed clear separation between all three groups, indicating that these three vibration conditions produced distinct brain proteomics profiles. Protein-protein interactions further revealed that identified proteins formed functional clusters associated with structural integrity, cell survival, and cell signalling (Tables 1–3). Collectively, these findings suggest that WBV at both frequencies, compared to the control, engages in biological pathways relevant to cognitive function, warranting further investigation in both animal models and human populations.

While vibration conditions altered protein expression in the brain proteome, the proteins altered in each condition differed. In 4 Hz conditions, differentially regulated proteins centred around structural/cytoskeletal remodelling and calcium handling [38,39]. Alterations in these functions could result in synaptic connectivity issues [41]. While 30 Hz conditions resulted in the differential regulation of proteins involved in axonal guidance, metabolic support, and stress response, possibly reflecting different adaptive or degenerative mechanisms. Notably, a prior study investigating low-intensity WBV at 30 Hz demonstrated improvements in spatial memory and a reduction in anxiety in rats, suggesting that specific molecular pathways, particularly those involved in axonal guidance, may underlie the effect of higher frequencies [42]. Given the distinct proteomics signatures of 4 Hz and 30 Hz in the current study, it is plausible that 4 Hz exposure could lead to different behaviours than those observed in that study [39].

Among the identified cognition-associated differentially regulated proteins were several with established roles in various cognitive processes. In 4 Hz exposure, plasma membrane calcium-transporting ATPase 3 (PMCA3), the 14-3-3 protein family, and Calcium/calmodulin-dependent protein kinase type II subunit beta (CaMKIIβ) were identified. PMCA3 is an essential pump involved in calcium regulation within neurons. Calcium regulation is vital for various neuronal functions, including signalling, neurotransmission, and synaptic maturation [38,43]. Calcium dysregulation and alterations to the PMCA protein family are linked to cognitive defects [41,43,44]. The 14-3-3 protein family, comprising several isoforms, is involved in various mental processes, including neural signalling, neuronal development, neuroprotection, and synaptic transmission. Alterations in this protein family induce cognitive decrements, including impairments in learning and memory [39,45]. In a prior study, CaMKIIβ was identified as an essential protein in hippocampal learning, plasticity, and long-term potentiation [46]. The differential regulation of these proteins suggests that 4 Hz WBV exposure influences calcium homeostasis, intracellular signalling, and synaptic plasticity, potentially disrupting biological pathways that control cognitive and behavioural function. Whether these factors contribute to disturbances in human performance in a vibration-intensive occupation (e.g. farming) is uncertain; however, they provide direction for further investigation into behavioural changes in both animal and human models.

Following 30 Hz exposure, notable differentially regulated proteins with established roles included the 14-3-3 protein family, microtubule-associated protein 1B (MAP1B), and synaptotagmin-2 (SYT2). Similar to 4 Hz conditions, various isoforms of 14-3-3 proteins were differentially regulated in 30 Hz conditions. MAP1B is involved in maintaining synapse integrity, promoting axon growth, and regulating neuronal function [37,47]. Most notably, MAP1B overexpression rescues memory in rodents [48]. As 30 Hz resulted in the overexpression of MAP1B, this may relate to cognitive improvements in response to this frequency. Another differentially regulated protein in response to 30 Hz stimulation was SYT2, a critical calcium sensor vital for fast neurotransmitter release, attention, and memory [49]. As 30 Hz has been shown to improve spatial memory in rats [42], it is plausible that the differential regulation witnessed in cognition-associated proteins in response to this frequency may function to conserve or promote cognition. The behavioural outcomes observed in response to 30 Hz WBV [42,48] may reflect that the proteomics changes observed at 30 Hz are suggestive of potentially adaptive or neuroprotective molecular signatures rather than neurodegenerative.

The observed proteomics changes under the 4 Hz and 30 Hz conditions may reflect several underlying biological mechanisms. Several proteins identified following 4 Hz exposure are involved in calcium handling [38,39]. Notably, the 4 Hz condition may induce elevated intracellular calcium, thereby prompting the downstream upregulation of proteins involved in calcium handling and regulation, such as CaMKIIβ and PMCA3 [50], which could influence synaptic plasticity and neuronal function [38,43,46]. In contrast, the 30 Hz condition resulted in a distinct proteomics profile, consisting of a balanced distribution of up- and down-regulated proteins. These proteins are associated with processes related to neuronal development, axonal guidance, and calcium-triggered neurotransmitter release, all of which are linked to neural functions such as synaptic remodelling and structural plasticity [51–53]. Alterations in these neural functions suggest that 30 Hz exposure could engage adaptation rather than overt damage. However, whether these proteomics alterations reflect a compensatory, neuroprotective, or harmful response remains unclear, as our study was not designed to measure those outcomes and warrants further investigation.

**Figure 6.**
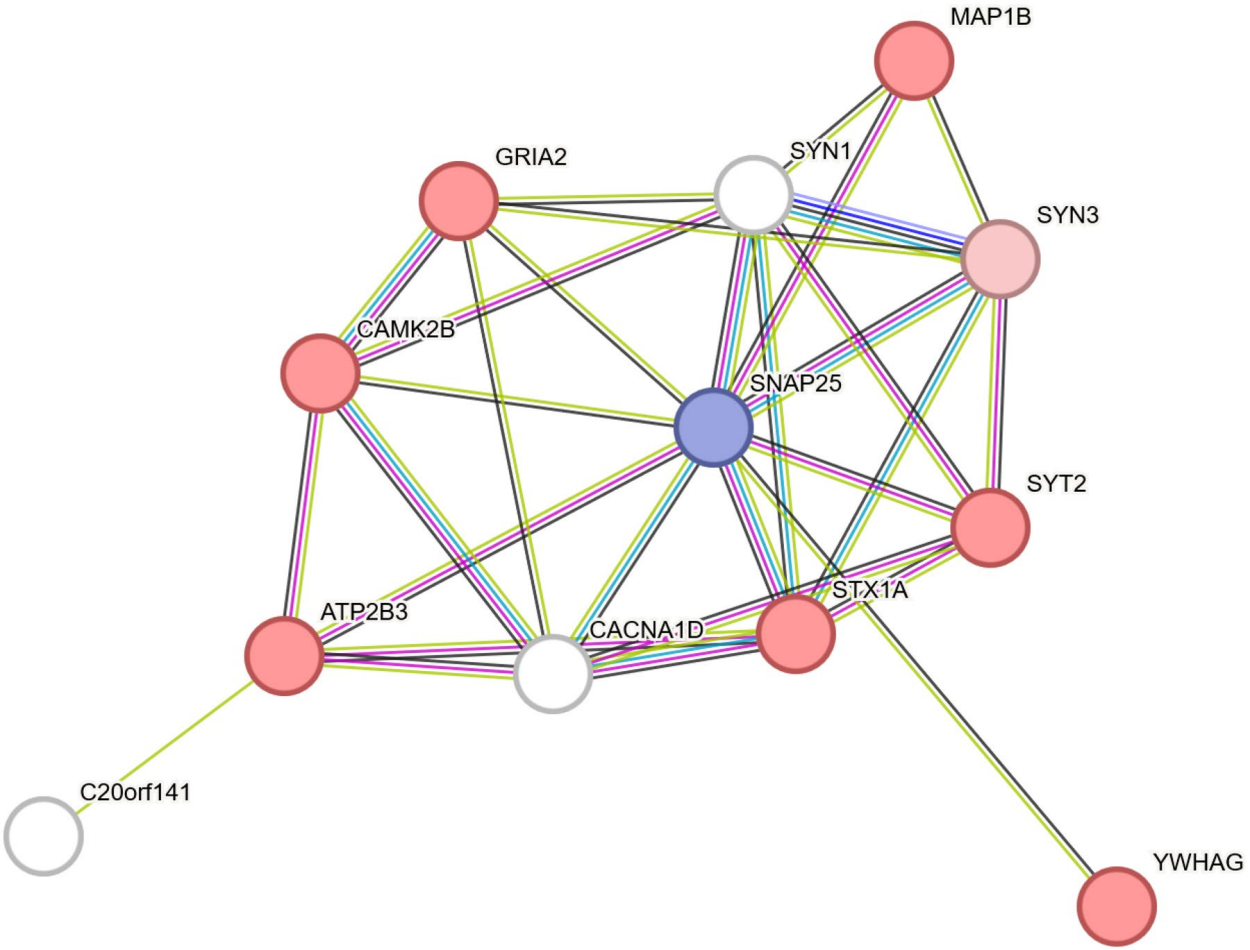
String analysis showing interaction network with SNAP25.

When comparing 30 Hz vs 4 Hz protein expressions, differentially regulated proteins included syntaxin-1A (STX1A), beta-synuclein (SNCB), and glutamate receptor A2 subunit (GluA2). Interestingly, STX1A was upregulated in the 4 Hz group compared to the control, but not in the 30 Hz group compared to the control. An increase in STX1A has been previously identified as a potential biomarker indicative of brain injury [54]. Thus, the upregulation of this protein in our 4 Hz experiment, but not in the 30 Hz, may also be demonstrating a response in the rat brain to a damaging insult. SNCB is a protein involved in synaptic plasticity, which is essential for learning and memory; thus, disruption of SNCB may impair cognitive function [40]. GluA2 is a critical component of glutamate receptors that facilitate synaptic transmission in neurons. Disruptions to GluA2 are linked to impairments in memory, spatial awareness, and synaptic plasticity [50]. These findings further suggest that the biological effects of WBV are frequency-dependent. The difference in frequency (30 Hz vs. 4 Hz) produced distinct proteomics signatures in the brain (Figure 3), underscoring how different WBV levels may affect unique biological pathways and functions. A further STRING analysis (Figure 6) reveals a strong interaction network between our proteins and SNAP25, which, in conjunction with STX1A, may indicate brain injury and disrupted synaptic function [54].

## Limitations

While our proteomics results are informative, we acknowledge that proteomics signatures alone cannot establish functional cognitive outcomes. As such, further research should integrate cognitive behavioural testing to explore the relevance of these findings. Another limitation of this study was the evaluation of stress in the animals. While rats were monitored for observable signs of stress, such as porphyrin staining and excessive grooming [48,49], no standardized tool was used to evaluate stress levels among rats. While both control and experimental rats were exposed to the same housing and restraint conditions, our design was unable to assess potential stress-by-vibration interactions, which could confound the observed proteomics results. A further limitation concerns the use of whole-brain tissue for proteomics analysis. While using whole-brain tissue enables global protein identification, it reduces anatomical specificity, potentially masking region-specific effects. Future studies addressing these limitations will strengthen the interpretation of proteomics findings in this context.

## Conclusions

In summary, our proteomics study in a rat model demonstrates that occupationally relevant WBV frequencies produce distinct, frequency-dependent alterations in the brain proteome, particularly within pathways related to synaptic plasticity, cytoskeletal organization, neurotransmission, and neuroprotection. The proteomics signatures identified in the 4 Hz and 30 Hz conditions differed significantly, suggesting that frequency variations can elicit distinct physiological responses. This study provides novel insights into an unexplored area of research on the cognitive effects of occupational WBV, using mass spectrometry-based proteomics, while further research integrating proteomics with behavioural testing should be aimed at exploring the relevance of these findings.

## Data Availability

The mass spectrometry data have been deposited to the ProteomeXchange consortium *via* the PRIDE partner repository with the data set identifier: PXD069749 [55]. Data will be made available upon reasonable request.
